# Perspectives on enhancing physical activity and diet for health promotion among at-risk urban UK South Asian communities: a qualitative study

**DOI:** 10.1136/bmjopen-2014-007317

**Published:** 2015-02-27

**Authors:** Laura Cross-Bardell, Tracey George, Mandeep Bhoday, Helena Tuomainen, Nadeem Qureshi, Joe Kai

**Affiliations:** Division of Primary Care, University of Nottingham, Nottingham, UK

**Keywords:** PUBLIC HEALTH, PRIMARY CARE, QUALITATIVE RESEARCH, SOCIAL MEDICINE

## Abstract

**Objectives:**

To explore perspectives on enhancing physical activity and diet among South Asians in urban deprived communities at high risk of chronic disease and to inform development of culturally appropriate health promotion intervention.

**Design:**

Qualitative study using semistructured one-to-one and family group interviews with thematic analysis of data.

**Setting:**

Urban disadvantaged communities in the East Midlands of the UK.

**Participants:**

45 respondents, including 34 people of South Asian origin (16 at-risk individuals, six family groups involving 18 relatives), of mainly Pakistani and Indian origin, including 16 non-English speakers; and 11 health professionals working locally with communities of concern.

**Results:**

South Asian participants underlined the challenges of requiring family members across generations to engage in modifying dietary behaviours, and the central role of communal eating of traditional ‘Asian’ food in their cultural lives. Barriers to increasing physical activity included cost, personal safety and lack of time outside of long working hours and carer commitments. However, increasing walking activity was regarded as feasible by both community and health professional participants. Respondents emphasised using a social approach for potential interventions, undertaking activity with family or friends and with bilingual community peers to facilitate engagement, motivation and support. Spoken content and delivery of interventions was favoured, including personal stories and multilingual audio–visual information; within local informal rather than provider settings, including the home; and aided by pedometers for self-monitoring.

**Conclusions:**

Focusing on physical activity by increasing walking may hold promise as health promotion in this deprived South Asian community context. Further intervention development, with exploration of feasibility and acceptability of the social approach and elements suggested, is merited.

**Strengths and limitations of this study**

- This study has emphasised sensitivity to the local cultural context to inform preliminary health promotion intervention development by gaining perspectives from both individuals and their families, including non-English speakers, from ‘harder to reach’ at-risk deprived South Asian communities about what health promotion may be appropriate and feasible for them.
- Information on use of theoretical models in health promotion interventions for South Asian communities is lacking. This study identifies the potential of using a social approach involving peer support to enhance engagement, motivation and behaviour change.
- Consultation with local stakeholders could have been more extensive with greater resource and time.
- This is helpful but preparatory work prior to developing and exploring intervention feasibility in practice, and before further experimental testing of effectiveness.

## Introduction

People of South Asian origin^i^ living in developed countries have considerably greater risk, morbidity and mortality from chronic diseases such as heart disease (CHD) and type II diabetes mellitus (T2DM) than European origin populations.^1–3^ The greatest opportunity to address this personal and societal burden is by preventing or delaying onset of disease in those often socially disadvantaged communities at highest risk. While genetic factors contribute, lifestyle figures strongly in the development of much chronic disease. In particular, physical inactivity, obesity and a diet high in fat and sugar are major risk factors for cardiovascular disease, diabetes and cancer.^4–7^ Cardiovascular protective behaviours are associated with a lower risk of all-cause mortality^8^ and important in primary and secondary prevention of chronic disease. Moreover, lifestyle interventions have been shown to prevent T2DM in adults at higher risk.^9^

Culture inevitably shapes attitudes and behaviours concerning physical activity and diet, with cross-cultural differences, including language, increasing the challenges of delivering appropriate interventions. In relation to obesity, South Asians in developed countries are not only particularly sensitive to the detrimental metabolic effects of adiposity,^10^ but also less physically active than European origin populations.^11^ With growing concern about child and adult obesity, evidence for the development and evaluation of culturally appropriate interventions tailored to the needs of high-risk South Asian communities is urgently needed.^12^

Evidence on physical activity and diet among South Asians is growing.^13–15^ However, a wide-ranging synthesis found insufficient evidence to make firm recommendations on health promotion interventions adapted for minority ethnic groups, in particular for improving diet or physical activity.^16^ A recent systematic review of pragmatic intervention studies with South Asians highlighted the need for prior qualitative research with target communities.^17^ This may yield insights that improve the relevance and acceptability of interventions,^17^ or help adapt existing interventions to specific community needs.^16^ ^17^ A recent trial with South Asians in Scotland used cross-cultural adaptation of a diabetes prevention intervention,^18^ and highlighted challenges in achieving the intended involvement of the wider family.^19^

The importance of developing appropriate interventions using an incremental approach, before evaluation of their effectiveness, is increasingly emphasised.^18^ The MRC framework for intervention development, for example, recommends reporting developmental phases in some detail.^20^ We aimed to develop a culturally appropriate health promotion intervention for people of South Asian origin at high risk of chronic disease, in urban disadvantaged settings, prior to evaluating feasibility in practice. In this paper, we report an initial phase of primary qualitative research to enable intervention development and its focus to be informed directly by those communities where intervention delivery was intended.

## Methods

### Sampling and data generation

We explored perceptions and experiences of strategies for enhancing physical activity and diet with a purposeful sample of people of South Asian origin living in the East Midlands, in localities ranked among the 20% most deprived nationally by Index of Multiple Deprivation.^20^

One-to-one semistructured interviews were conducted with 16 individuals at high risk of diabetes and other chronic disease (overweight or obese and/or a family history of diabetes in a first-degree relative). Participants were identified by two local general practices with 85% of their patients from South Asian inner city populations. In parallel, six family group interviews were undertaken involving 18 household members or relatives of these individuals to discuss and understand the wider family's potential role in supporting changes in physical activity or diet.

Interviews were conducted in the participants’ own homes by a team of three researchers (including those of female Muslim, Sikh and bilingual Punjabi/Urdu speaking backgrounds). They explored attitudes and views on realistic and relevant health promotion, and what might be helpful and effective from their experience of undertaking or discussing lifestyle change. This included seeking views on examples of published interventions, and how interventions might be facilitated for their context. Over a third of respondents were interviewed in Punjabi or Urdu. Most participants were overweight or obese, with no formal educational qualifications beyond school level. Their characteristics are summarised in table 1.

**Table 1:** South Asian participant characteristics

	n=34
Mean age in years (range)	41 (19–67)
Gender	
Female	23
Male	11
Self-defined ethnicity	
Pakistani	17
Indian	13
Bangladeshi/other South Asian	4
Religion	
Muslim	21
Sikh	11
Hindu	2
Formal educational attainment*	
No formal qualifications	9
School level—GCSE (16 year)	7
A level (18-year)/GNVQ equivalent	13
University level	4
Mean BMI (range)	28 (16–41)
Family history of diabetes in a first-degree relative	19
Preferred language	
English	19
Punjabi	7
Urdu	8

*Information on education not provided by 1 participant.

BMI, body mass index; GCSE, General Certificate of Secondary Education; GNVQ, General National Vocational Qualification.

In parallel, we conducted a focus group discussion with local providers and stakeholders working with these communities in inner city Derby and Nottingham. We explored views and experiences of health promotion provision and supporting lifestyle change in this local context. They included bilingual South Asian dieticians (2), bilingual GPs in practices with majority South Asian populations (2), local authority exercise officers (2), community health promotion workers (2), a public health consultant, and local health service commissioners (2).

All interviews were audiotaped and transcribed verbatim, with those in Punjabi/Urdu translated into English by independent translation services. Transcripts were then rechecked by a bilingual researcher for equivalence of meaning with the original audiotapes. Data were analysed thematically in parallel with data generation until saturation. Emerging themes were reviewed and discussed regularly by the multidisciplinary research team with backgrounds in social science, nursing, health research and primary care clinical practice.

## Findings

South Asian participants mostly recognised their diets and physical activity could be improved, and highlighted a range of barriers. Participants sought multilingual support and involvement of peers, both as advisors and in undertaking health promotion with others. They wanted an intervention with an emphasis on spoken communication, using personal stories, with peer support. Walking was identified as the most feasible and culturally appropriate physical activity that had the potential to include a social element and involve friends and family. They underlined the importance of relationships with peers, local and informal settings they could easily access, and activities that fitted around family life. Local health professionals echoed many of these community perceptions, and highlighted underinvestment of policy and services in supporting prevention of disease, in contrast to management of existing specific diseases.

### Perceptions and challenges of lifestyle change

Respondents underlined that communal eating of traditional ‘Asian’ food was central to their social and cultural lives, and key to their standing, particularly concerning hospitality with others. They further discussed dietary considerations and the difficulties of making and maintaining dietary changes as embedded within their household. Each household member had influence over food choices for everyone, which made changes difficult to implement when not everyone wanted to alter their diet. Participants spoke of commonly acquiescing to older relatives, in-laws and other family members’ preferences. Even when a family in a household had agreed to make changes, maintenance of dietary modification could be hindered by visits from friends or relatives whose preferences were adhered to. Experience of professional dietary advice could also be perceived as culturally inappropriate (box 1).

Box 1 **Challenges of dietary change**

*In Asian families, nobody’s going to be cooking for each individual, do you see what I mean? So...it’s rather difficult to take care of your diet...because it revolves around other people as well and not just you.* (Woman, 34y, Pakistani Muslim)

*When you live in a joint family it is different, it is very difficult. (...) people say ‘Well why do we have to eat this?’.....they just want to eat the normal (traditional) stuff, the regular stuff.* (Woman, Pakistani, Muslim, 32, Multigenerational household)

*(We) put extra couple of spoons of ghee in it for taste…That is all bad, but when you are used to that taste then it is difficult. (My) friend who is 10 years older to me, says if he doesn't eat Indian food then his body doesn't get lubrication...*(Man, 56y, Indian, Sikh)

*P3: Yes. There is not much support out there for somebody who's… I mean okay she says we're all going to go for a walk. If you do something seriously like food wise or exercise wise then there's not much support for you out there in our community. P2: And especially food wise, if you want to have boiled vegetables, jacket potato, you'd have to make that separate. P3: I do do that. P2: The rest of the family would not want to eat it. It's tasteless or… P1: Old people do that… P2: It's old people. My dad doesn't want to eat food like that, and my husband is from abroad and he doesn't want to eat that. But the rest of us are happy with that, but then we have to make those separate meals and its time consuming and you just end up giving up.* (Family group (P1) 52y, (P2) 26y and (P3) 32y, Pakistani women, Muslim)

*The one who advises on food...what do you call her...dietician… She comes to the doctors and advises us on what we should eat, what we shouldn't, but we lot are used to our vegetables and daal, .. Also they advise that we should use only one spoonful of ghee/olive oil/oil, only one spoon. When we cook we cook for the whole family, would that get cooked in one spoonful of oil? At least one whole ladle full will be used!* (Woman, 67y, Indian, Sikh)

A wider range of barriers to physical activity were described. Issues of personal safety, cost, family and childcare commitments and availability of free time away from long working hours were recurring concerns, in addition to the weather. Participants also identified some facilities as relevant to other communities rather than themselves (box 2).

Box 2 **Difficulties increasing physical activity**

*I think it's more do it in the summer because it is lighter and that. You know in the dark those people around the areas—you don't want to go out really. And I wouldn't want her [wife] to go out on her own walking the streets.*’ (Man, 66y, Indian, Sikh)

*I used to do it (exercise) when I went to secondary school. I had the option to do it and it's free. Now everything is costing me too much.* (Woman, 19y, Bangladeshi, Muslim)

*...plus, I think financially, you know, when you're not working, you can't afford membership for [name of gym], can you?* (Man, 44y, Pakistani, Muslim)

*I work 2 till 10pm and then I come back home and then I cook something or end up sleeping at 1, when am I supposed to use the treadmill?* (Woman, 24y, Pakistani, Muslim)

*I think especially when you live in a joint family it is different, it is very difficult. It's difficult to go out and join the gym and do what you want to because it's difficult enough to get that time for yourself anyway, .. it's a case of the family...*(Woman, 32y, Pakistani, Muslim)

*I mean we do it (walking) in summer, but in winter, there is the odd few that still go even though it is cold or whatever. I think we only do it June, July and August time and that's it*. (Woman, 32y, Pakistani, Muslim)

*I used to go on a bike ride ..jogging—that sort of stuff. Since we had children you don’t have as much time—there’s a lot of commitments with the kids, so that’s what’s changed.* (Man, 42y, Indian, Hindu)

*But they (gyms and swimming pools) are for the English, not for us.* (Man, 50y, Pakistani, Muslim)

### Preference for spoken information and experience

Participants sought health promotion information in a variety of languages but, even when translated appropriately, expressed frustration about receiving this in written forms. They highlighted variable levels of literacy in their communities. They also perceived that taking time to discuss issues in person ‘trumped’ receiving a leaflet. They sought information that was relevant, tailored to them and, ideally, based on personal experiences, rather than limited to facts about the health benefits of physical activity and a healthy diet (box 3).

Box 3 **Spoken information and experience**

*They (services) think, ‘oh OK, so we have to translate everything’...it doesn't automatically follow that you can read it, just because you can speak it. Just because you can understand it, doesn't mean you can read it. So you know...it has to be verbal*. (Woman, 35y, Pakistani, Muslim)

*Actually ‘talking’ because literature doesn't go very far...He [husband] wouldn’t, he can’t read. Even if he could, I don’t think he would really bother...it’s a case of actually sitting somebody down and talking and giving an example...*(Woman, 42y, Pakistani, Muslim)

*The problem is, is if you've got somebody at the end of the phone you can put it down...(but)...you'd make that little bit of an effort because you're face to face with somebody and I always prefer seeing somebody.* (Woman, 33y, Indian, Sikh)

*It would be better if it is someone who has experience themselves. Like my mum, as she was overweight, and...how she controlled it. If you show someone like that in a video people can look to them...and how she worked it out and this is how she lost weight....And it is someone who has been through it. If someone has been through it and they tell them. I think it is better.* (Woman, 27y, Pakistani, Muslim)

### Peer facilitation and motivation

Respondents sought in-person, face-to-face support to help them with lifestyle change. In particular, they shared enthusiasm for facilitation and motivation from peers in their community, rather than advice from health or other providers alone. The potential for any activity to include social elements was also important, as were the motivational effects of sharing attempts at health promoting activity with their family and friends. In this context, some participants had positively experienced taking up walking with family and friends as health promotion, or anticipated that this would be welcome in any intervention promoting walking (box 4).

Box 4 **Peer facilitation and motivation**

*I would prefer it to be somebody I know—I'd be more comfortable if it's somebody I know and then maybe—if it's somebody from the community—somebody who's in the same kind of environment as me, that will help*. (Woman, 33y, Pakistani, Muslim)

*You can share ideas and your worries with your peers...and, you know, discuss how they’ve done things differently*. (Man, 37y, Pakistani, Muslim)

*They probably just organise or just friends or even family members, they seem to go for a walk there (local park area) together, which you know, it seems to work and develop into a pattern. Some people are doing it, erm, and they're probably encouraging other people as well.* (Man, 53y, Indian, Sikh)

*So we thought if one person does it (more walking), if we all do it, it motivates that person as well. So we all do it to participate and help (mother and father with diabetes) as well*. (Woman, 27 y, Pakistani, Muslim, family group interview)

### Informal home-based setting

Participants favoured an informal rather than a more formal service or sports leisure settings for intervention delivery. Many suggested their homes, perceiving advantages for their cultural access and engagement, and reducing barriers to initiate or resource travel in terms of time or cost. There were mixed views regarding local faith and community centres as a setting and few participants suggested attending interventions in health provider settings (box 5).

Box 5 **Preference for a home setting**

*It’s probably ideal for somebody like me that someone is coming in, actually seeing you in person and they know that they’ve come to your household for you to get more attention that way. Yes, definitely, I could see myself liking that...because it is taking place in your own house – probably more comfortable as well.* (Woman, 25y, Pakistani, Muslim)

*You would listen to somebody who’s come to your house. You might miss it if someone throws a leaflet. But if someone comes to a house, explains to you, even if you’re sat down for face minutes and says look, will you please try this, it’s for your own benefit.* (Woman, 32y, Indian, Sikh)

*The thing about the community centre is that...there will be a lot of talking, and also there will be many people asking questions, and they may not be understood. So I think someone coming to your house is better.* (Male, 50y, Pakistani, Muslim)

*...like it takes up time, going to gym and coming back from gym as well, so if you are in your own house....that will save me time and I can do my other things as well with that.’* (Woman, 33y, Pakistani, Muslim)

*Rather than go to the gym, I prefer to do it at home.* (Woman, 61y, Indian, Sikh)

*P2: Yeah mom says she'd prefer if someone came home. Over the phone it's difficult for mum to understand.* *P1: I find it difficult to explain. Maybe I can explain myself better face to face and you may be able to understand me. On the phone I tend to mix up my past and future. Like this is not difficult.’* *P2: In person at home would be easy for her mother to understand than* via *the phone*. (Woman, 26y (P2) and 52y (P1), Pakistani, Muslim)

### Walking and pedometers

Walking was most commonly considered to be feasible, culturally appropriate and accessible physical activity by participants. South Asian respondents were keen on the idea of being supported to increase their walking. They spoke of how this could be performed with peers socially and for motivation, the advantages if offered addressing practical concerns such as safety and cost, and exploiting these in their day-to-day activities or in local parks. Some participants had previous positive experience of using pedometers, highlighting their use for self-monitoring and motivation. Others similarly perceived their potential use as helpful in setting walking goals. These perceptions were echoed by local health providers (box 6).

Box 6 **Feasibility of walking and use of pedometers**

‘*It's (walking) cheaper and doesn't cost you anything.’* (Man, 44, Pakistani, Muslim)

*You feel scared going alone (out walking). If there are other women then you feel safe and don't feel scared.* (Woman, 52, Pakistani Muslim)

*Not on my own. Nowadays, I would love walking but it’s a fear isn’t it—you live in this a funny old world. You don’t know really. It’s not very safe, but when you are together at least if you are in [local area] or somewhere else—the park, you know you are together.’* (Woman, 62y, Indian, Sikh)

*If someone came to your house and said this number of steps would be really ideal, so you’ve got a target, and you were provided with one of them (pedometer)....You’d just think generally, when I’m walking, I’ve done this much steps, it might motivate you because you would just think—I’ve met my target for today. I’ve done such and such an amount of steps.* (Woman, 25y, Pakistani, Muslim)

*I used it (pedometer) for some time, just to see how much I was walking. I was doing a lot of walking at work...but not as much as I thought. You know, so it showed me that I was doing it, compared to what I was doing before… I would probably look at it, ‘I've not done much walking at all’ but I get up and go and do some more...(*Man, 53y, Indian, Sikh)

*Walking...doesn’t cost anything. Even though it's inner city, we’ve got two or three parks within a few minutes’ walk.* (Health Improvement Principal)

*If you actually do go to the parks here in the morning or the evening, there are a lot of South Asian people now with their trainers on and walking. So I think the messages are getting through.* (General Practitioner)

‘*To encourage so if there was a few people from let's say the Asian community in one area they started walking. The other people around them so word of mouth will get more and more. A problem with our community is that men and ladies don’t like to walk together so start two different clubs, one for women and one for men.’* (Man, 38y, Pakistani, Muslim)

‘*And once they fix them onto you, you’d just think generally, when I’m walking, I’ve done this much steps, it might motivate you because you would just think—I’ve met my target for today. I’ve done such and such an amount of steps.’* (Woman, 25y, Pakistani, Muslim)

### Lack of support for prevention

Local health providers highlighted the underinvestment of both health policy and service resources in supporting the prevention of disease in contrast to care and management of disease once diagnosed. They found this frustrating and were supportive of access to better relevant provision. They noted lack of systematic approaches or referral pathways for primary prevention, which thus became haphazard in day-to-day practice (box 7).

Box 7 **Underinvestment in prevention**

*....(Yes) if I was to visit my GP and they said ‘you are at risk from developing diabetes, you need to do more exercise’ you’d get a bit of help and advice there but that seems to be pretty much as far as it goes. You get a leaflet. But I come back in six months time with developed diabetes then all of a sudden it is like: “now I can refer you on now—to me that just seems a bit unfair”* (Exercise Referral Officer)

*...As a first point of contact for most patients, prevention is very* ad hoc*, depending on the interest of practitioners...there’s no financial resource for practices to engage in primary prevention...we don’t have clear protocols or referral pathways so it is all* ad hoc*.* (General practitioner)

*(We do) a one-off two hour (health promotion) session because at the moment that’s all our resources will enable us to do...Unfortunately with more (diabetes) services being commissioned, they’ve put prevention on the back burner. Which is very sad, really.* (Health Improvement Principal)

*If we see patients say with impaired glucose tolerance (at risk of diabetes) at the moment there is no clear pathway for them...So we give them some basic advice (about exercise and weight) and say ‘see you next year and we’ll do another fasting glucose’* (General practitioner)

## Discussion

This study has identified qualitative insights about health promotion perceived as acceptable and feasible by people from urban South Asian communities at risk of chronic disease. The findings suggest that use of the following will be relevant to intervention development: a focus on enhancing physical activity by increasing walking; use of pedometers; multilingual spoken content and delivery, including use of personal experiences; peer support, for example by bilingual community members, to facilitate engagement and motivation, and undertaking activity with family or friends; and delivery in local informal settings, including the home.

Strengths of the work include obtaining both individual and family perspectives, and actively including non-English speakers, from ‘hard to reach’ urban disadvantaged South Asian populations. Field researchers shared languages and religious backgrounds with participants. This may have enhanced access to and authenticity of data, in addition to avoiding the need for third party translators. Consultation with target communities, prior to intervention development or use, is relatively unusual but offers the advantages of potentially improving intervention sensitivity and acceptability. These qualitative findings must be interpreted with regard to the study context and sample as described. The study was pragmatic and commissioned to expeditiously progress to further work locally. We recognise that aspects, such as consultation with other community stakeholders, could have been more extensive with greater resources and time. We intend, for example, to engage local faith centres in further exploring intervention development.

The current findings are generally consistent with existing qualitative research with South Asians in the UK, but offer additional insights for intervention development using a social approach. Physical activity has been positively perceived as beneficial for health in these communities,^21–25^ though its links to prevention of disease were less well recognised.^17^ Barriers to engaging in physical activity, such as women's roles in caring for family, physical modesty, safety and unmet information needs, have been identified.^14^ ^21–24^ ^26–28^ Similar to ‘majority’ populations, other barriers include lack of motivation, fear of symptoms during exercise and lack of time and money.^14^ ^15^ ^21^ ^22^ ^26^ As in the current study, the major challenges of changing cultural dietary practices have been emphasised, in particular the importance of sharing of traditional food, commonly high in fat and sugar, to maintaining social relationships and cultural identity in South Asians;^15^ ^24^ ^29^ and, similarly for example, adhering to the recommended diet being the most difficult part of living with diabetes.^30^

Others have found South Asian access to health promotion to be hindered by language, and the failure of health providers to offer practical advice on diet, physical activity and reducing the risk of disease.^14^ ^28^ This might chime with the lack of support for primary prevention which frustrated local providers in this study.

This study identifies the potential of using a social approach, consistent with social learning and cognitive theory,^31^ involving peer support to enhance engagement, motivation and behaviour change. Our findings suggest the importance of sociability and interaction, and a feeling of being among people such as oneself. Engagement at the wider levels of family or community in relation to physical activity, as suggested here, might also assuage concerns about propriety and modesty, particularly for some women, as noted in a previous work.^21^ Given the unfamiliarity of ‘exercise’ as a formal activity and discomfort with mixed-sex sports and leisure provision for some South Asians,^14^ ^21–24^ ^26–28^ a walking intervention, delivered and promoted in familiar settings such as the home and local parks, might offer greater prospect of success than those in more formal healthcare environments. Bilingual community peer facilitators may be valuable in supporting this,^17^ especially when the inner city South Asian communities of concern may not be easily reached through formal planned activities but rather more informal social contacts. There is also good evidence that the techniques highlighted by some of our participants, such as goal setting and self-monitoring, are effective in changing behaviour.^32^

Recent studies suggest the potential of interventions to increase walking in those at risk of chronic disease,^33^ ^34^ but have not focused on deprived South Asian populations. For our participants, increasing their walking appeared achievable and the most realistic focus for starting to improve their health and reduce risk of disease. We are proceeding to explore the feasibility and acceptability of community peer facilitated intervention to increase walking, using a social approach in informal settings, with target inner city South Asian communities.

## Footnotes

i The term ‘South Asian’ in this paper refers to those born in, or descended from those born in the Indian subcontinent including Pakistan, India, Bangladesh and Sri Lanka.
